# An *In Vitro* Study of the Antimicrobial Effects of Indigo Naturalis Prepared from *Strobilanthes formosanus* Moore

**DOI:** 10.3390/molecules181114381

**Published:** 2013-11-21

**Authors:** Yin-Ru Chiang, Ann Li, Yann-Lii Leu, Jia-You Fang, Yin-Ku Lin

**Affiliations:** 1 Biodiversity Research Center, Academia Sinica, Taipei 115, Taiwan; E-Mail: yinru915@gate.sinica.edu.tw; 2 Graduate Institute of Natural Products, College of Medicine, Chang Gung University, Taoyuan 333, Taiwan; E-Mails: tommy19880726@gate.sinica.edu.tw (A.L.); ylleu@mail.cgu.edu.tw (Y.-L.L.); 3 Research Center for Industry of Human Ecology, Chang Gung University of Science and Technology, Taoyuan 333, Taiwan; E-Mail: fajy@mail.cgu.edu.tw; 4 Department of Traditional Chinese Medicine, Chang Gung Memorial Hospital, Keelung 204, Taiwan; 5 School of Traditional Chinese Medicine, Chang Gung University, Taoyuan 303, Taiwan

**Keywords:** indigo naturalis, *Strobilanthes formosanus* Moore, tryptanthrin, isatin, nail psoriasis, onychomycosis

## Abstract

Indigo naturalis is effective in treating nail psoriasis coexisting with microorganism infections. This study examines the antimicrobial effects of indigo naturalis prepared from *Strobilanthes formosanus* Moore. Eight bacterial and seven fungal strains were assayed using the agar diffusion method to examine the effects of indigo naturalis and its bioactive compounds. The bioactive compounds of indigo naturalis were purified sequentially using GFC, TLC, and HPLC. Their structures were identified using mass spectrometry and NMR spectroscopy. UPLC-MS/MS was applied to compare the metabolome profiles of indigo naturalis ethyl-acetate (EA) extract and its source plant, *Strobilanthes formosanus* Moore. The results of *in vitro* antimicrobial assays showed that indigo naturalis EA-extract significantly (≥1 mg/disc) inhibits Gram-positive bacteria (*Staphylococcus aureus*, *S. epidermis* and methicillin-resistant *S. aureus* (MRSA)) and mildly inhibits non-dermatophytic onychomycosis pathogens (*Aspergillus fumigates* and *Candida albicans*), but has little effect on dermatophyes*.* Isatin and tryptanthrin were identified as the bioactive compounds of indigo naturalis using *S. aureus* and *S. epidermis* as the bioassay model. Both bioactive ingredients had no effect on all tested fungi. In summary, indigo naturalis prepared from *Strobilanthes* *formosanus* Moore exhibits antimicrobial effects on *Staphylococcus* and non-dermatophytic onychomycosis pathogens. Tryptanthrin and isatin may be its major bioactive ingredients against *Staphylococcus* and the inhibitory effect on MRSA may be due to other unidentified ingredients.

## 1. Introduction

Indigo naturalis (or Qing Dai in Chinese) is a dark-blue powder originating from the leaves and branches of various indigo-producing plants such as *Baphicacanthus cusia* (Nees) Bremek, *Indigofera tinctoria* L., *Isatis indigotica* Fort, *Polygonum tinctoria* Ait, and *Strobilanthes formosanus* Moore. Indigo naturalis has been used as a textile dye, paint pigment and medicine in China and India for many centuries. In traditional Chinese medicine (TCM), indigo naturalis has been used commonly for treating various infectious and inflammatory skin diseases. Modern research has revealed that indigo naturalis is useful for antipyretic, anti-inflammatory, antiviral, antimicrobial, anticancer and detoxifying purposes [1,2].

We have used indigo naturalis to treat more than 10,000 patients with psoriasis during the past 11 years. Our clinical trials showed that topical application of indigo naturalis significantly improved skin psoriasis [3,4]. In the past 5 years, indigo naturalis extract in oil (Lindioil) has been used to treat nail psoriasis and a preliminary non-controlled trial found such a treatment to be effective [5,6]. In our previous observations, topical indigo naturalis can ameliorate psoriatic nails coexisting with fungus and bacterial infection, such as onychomycosis and chronic paronychia.

Nail psoriasis is a notoriously difficult disease to treat and has the following characteristics: nail dystrophy, thickening, loss of luster, raising, changes in color, and friability. Dystrophic nails lose their natural protective barrier and are, therefore predisposed to fungal infections [7]. People with nail psoriasis have a higher incidence of onychomycosis, which is the most common nail disease worldwide, constituting almost half of all onychopathies [8].

The causative pathogens in onychomycosis include dermatophytes, *Candida* species, and non-dermatophytic molds. Dermatophyes are the most common pathogens that infect the nail apparatus. Among them, *Trichophyton rubrum* is the most common, and *Epidermophyton floccosum*, *Trichophyton violaceum*, *Microsporum gypseum* and *Trichophyton tonsurans* are less common [9]. On the other hand, onychomycosis caused by *Candida* spp. and non-dermatophytic molds (such as *Aspergillus* spp*.*) represents a higher incidence in the tropics and subtropics with a hot and humid climate, such as Taiwan [9].

Paronychia is an inflammation of the structure surrounding the nails. Whether acute or chronic, paronychia is often associated with onycholysis and/or nail plate dystrophy which results in a breakdown of the protective barrier between the nail and the nail fold. The introduction of organisms into the moist nail crevice results in bacterial or fungal (yeast or mold) colonization of the area. Paronychia results from multi-microbial infections, with the presence of both aerobic and anaerobic bacteria in about three-fourths of the cases. Typically *Staphylococcus aureus*, *Streptococcus* spp*.*, *Klebsiella pneumoniae*, *Pseudomonas aeruginosa*, and *Candida albicans* are the predominant pathogens [10]. *S. aureus* and *C. albicans* cause soft tissue infection of the nail fold, which often induces painful acute inflammation in chronic paronychia. *Pseudomonas* may colonize the dystrophic nail plate, causing a greenish discoloration [11].

Although clinical observations have indicated that indigo naturalis can ameliorate psoriatic nail coexisting with onychomycosis and paronychia, it is still unknown if the topical therapy directly inhibits the growth of pathogens on the nails and/or prevents the invasion of pathogens by restoring the damaged nail structure. The aim of this study is to investigate whether indigo naturalis possesses antimicrobial effects on the pathogens of skin and nail infections. The active compounds of indigo naturalis were purified and identified. In addition, a metabolomic approach was applied to elucidate the origin of its bioactive compounds.

## 2. Results and Discussion

### 2.1. Antimicrobial Activities of Indigo Naturalis EA-Extract

In this study, an agar diffusion assay was utilized to assess the antimicrobial effect of the indigo naturalis EA-extract (in the range between 0~4 mg per disc) because: (1) some tested microorganisms, especially fungal species, were unable to grow evenly in liquid media; (2) the color of indigo naturalis extract was dark-blue. These conditions made a microtiter plate assay unsuitable for this study.

Five strains of Gram-positive bacteria, three strains of Gram-negative bacteria, and seven strains of fungi were used in the antimicrobial assay (Table 1). Among them, *S. aureus* ATCC 6538, Methicillin-resistant *S.* (MRSA) ATCC 43,300, *S. pneumoniae* ATCC 33,400, *K. pneumoniae* ATCC 13,883, and *P. aeruginosa* ATCC 27853 are pathogens in humans. In addition, *S. epidermis* ATCC 12,228 is an opportunistic pathogen on skin.

**Table 1:** Microbial strains assayed for antimicrobial effects.

Microbial strains	Growth temperature (°C)	Media used for cultivation
*Gram-positive bacteria*		
*Bacillus subtilis* ATCC 21,778	30	ATCC medium 3
*S. aureus* ATCC 6538	37	ATCC medium 18
MRSA ATCC 43,300	37	ATCC medium 18
*S. epidermis* ATCC 12,228	37	ATCC medium 3
*S. pneumoniae* ATCC 33,400	37	ATCC medium 260
*Gram-negative bacteria*		
*Escherichia coli* ATCC 23,815	37	ATCC medium 3
*K. pneumoniae* ATCC 13,883	37	ATCC medium 3
*P. aeruginosa* ATCC 27,853	37	ATCC medium 18
*Fungi*		
*Aspergillus fumigates* ATCC 1022	26	ATCC medium 325
*C. albicans* ATCC 18,804	25	ATCC medium 200
*Cryptococcus neoformans* ATCC 13,690	26	ATCC medium 200
*Saccharomyces cerevisiae* ATCC 26,603	30	ATCC medium 200
*E. floccosum* ATCC 18,397	30	ATCC medium 28
*M. gypseum* ATCC 14,683	26	ATCC medium 28
*T. rubrum* ATCC 10,218	26	ATCC medium 2166

Table 2 shows the growth inhibitory activities of the indigo naturalis EA-extract against tested bacteria. Results revealed that indigo naturalis EA-extract (≥1 mg/disc) had inhibitory effects against *S. aureus*, *S. epidermis* and MRSA. Nevertheless, there was no inhibitory effect found for other Gram-positive and Gram-negative species in the presence of indigo naturalis EA-extract (<4 mg per disc). Moreover, the inhibitory activities of indigo naturalis EA-extract against the above three *Staphlycoccus* strains varied in a dose-dependent manner (Table 2).

**Table 2:** The antimicrobial effects of indigo naturalis EA-extract on tested microorganisms. Only the strains sensitive to the indigo naturalis EA-extract are shown.

Test Strains	Quantity of indigo naturalis EA-extract per disc (mg)				
	0 mg	1 mg	2 mg	3 mg	4 mg
*Gram-positive bacteria*					
*S. aureus*	0 ± 0 *	138 ± 18	157 ± 23	174 ± 17	188 ± 24
MRSA	0 ± 0	167 ± 13	198 ± 18	209 ± 25	225 ± 38
*S. epidermis*	0 ± 0	77 ± 8	86 ± 5	95 ± 13	108 ± 24
*Fungi*					
*A. fumigates*	0 ± 0	39 ± 8	51 ± 9	68 ± 15	79 ± 13
*C. albicans*	0 ± 0	31 ± 5	43 ± 7	48 ± 11	53 ± 10
*C. neoformans*	0 ± 0	0 ± 0	0 ± 0	0 ± 0	18 ± 4
*S. cerevisiae*	0 ± 0	0 ± 0	0 ± 0	24 ± 9	28 ± 12
*E. floccosum*	0 ± 0	0 ± 0	0 ± 0	0 ± 0	9 ± 3
*T. rubrum*	0 ± 0	0 ± 0	0 ± 0	0 ± 0	14 ± 5

* The diameters of the inhibition zones (diameter of inhibition zone minus diameter of disc) were measured in mm after incubation for 24 h at the optimal temperature for the individual strains tested (see Table 1). Data are the means of triplicates ± standard deviation of a representative experiment.

The antifungal effects of indigo naturalis EA-extract were assayed in the following fungal species: *T. rubrum* ATCC 10,218, *M. gypseum* ATCC 144,683, *E. floccosum* ATCC 18,397, *C. albicans*, *A. fumigates* and *C. neoformans*. Results showed *A. fumigates* and *C. albicans* were mildly inhibited by indigo naturalis EA-extract in a dose-dependent manner (Table 2). Furthermore, indigo naturalis EA-extract also showed a weak inhibitory effect on *S. cerevisiae* and little, if any, effect on the other three tested dermatophytes (Table 2).

The results of the data indicate that indigo naturalis EA-extract has inhibitory effects on *S. aureus*, MRSA and *S. epidermis* even in dosages as low as 1 mg/disc*.* This evidence is in agreement with TCM clinical experience in the treatment of infectious bacterial skin diseases. In TCM, indigo naturalis has been used for centuries to remedy abscesses, carbuncles, furuncles and cellulitis.

### 2.2. Purification and Identification of the Antibacterial Compounds of Indigo Naturalis

**Figure 1:**
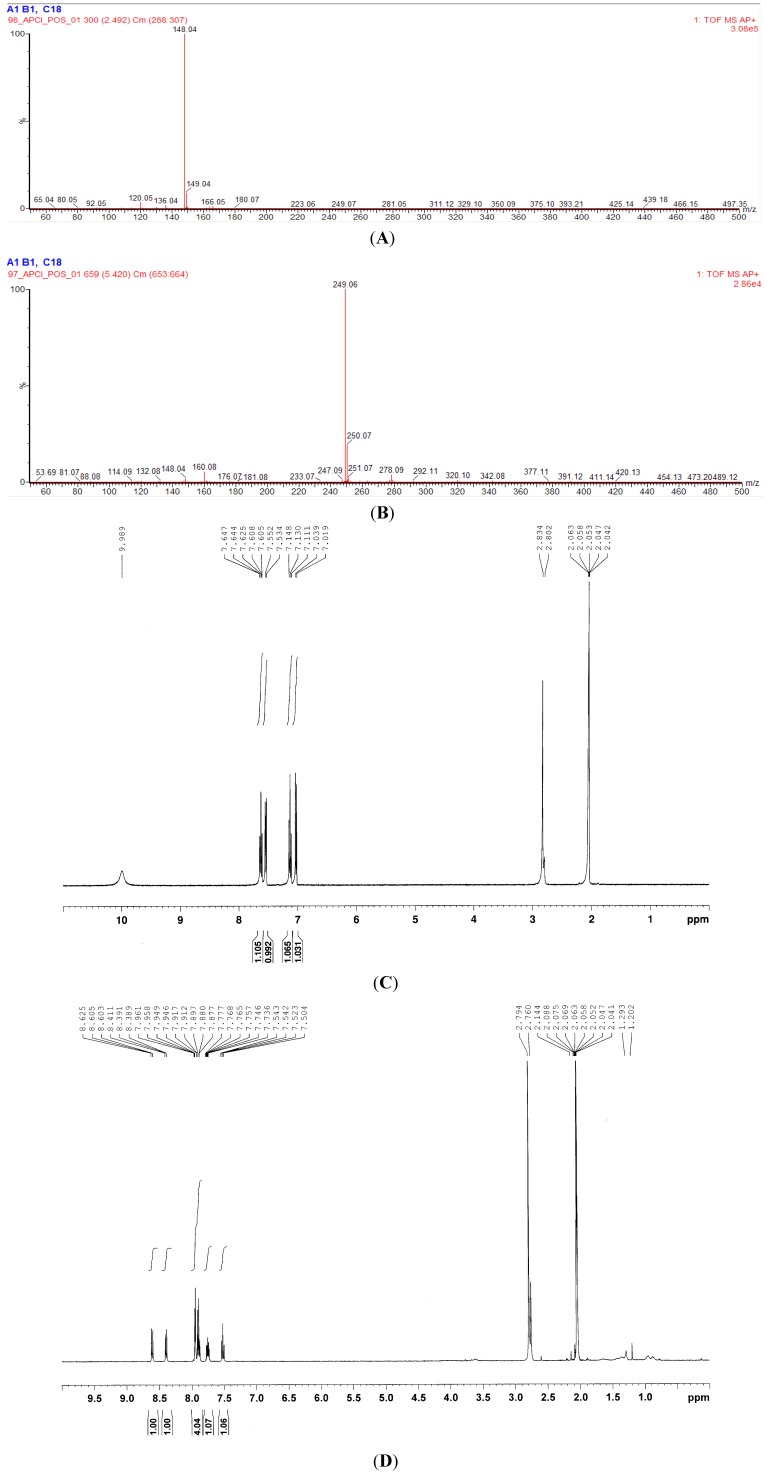
APCI-MS and ^1^H-NMR spectra of the anti-*Staphylococcus* compounds (IN-1 and IN-2) purified from the EA-extract of indigo naturalis; (**A**) APCI-mass spectrum of the compound IN-1; (**B**) APCI-mass spectrum of the compound IN-2; (**C**) ^1^H-NMR spectrum of the compound IN-1 (400 MHz, acetone-*d*_6_); (**D**) ^1^H-NMR spectrum of the compound IN-2 (400 MHz, acetone-*d*_6_).

Gel permeation, thin-layer, and high performance liquid chromatographies (HPLC) were applied in sequence to purify the active compounds from indigo naturalis EA-extract. We applied a bioassay-guided strategy to trace the active compounds present in the indigo naturalis EA-extract. Agar diffusion assay was used to avoid the optical disturbance of active fractions. Indigo naturalis EA-extract showed apparent inhibitory effects on the growth of *Staphylococcus* members *S. aureus* ATCC 6538 and *S. epidermis* ATCC 12,228 (see Table 2); therefore, these were used as the model organisms in the bioassays. A total of 200 g of indigo naturalis powder was used and, after purification procedures, two active compounds (IN-1, 2 mg; IN-2, 5 mg) were isolated which were respectively identified as isatin and tryptanthrin. These compounds were identified by comparing their NMR and APCI-mass spectroscopic data (Figure 1A–D) with those of standard compounds purchased from Sigma-Aldrich Chemicals.

### 2.3. Antimicrobial Activities of the Bioactive Compounds of Indigo Naturalis

The antimicrobial activities of the bioactive compounds of indigo naturalis, isatin and tryptanthrin, were assayed against a panel of microorganisms using agar diffusion assay. Table 3 and Table 4 summarize the results, which are highly coincident to those of indigo naturalis EA-extract. This phenomenon suggests that isatin and tryptanthrin are the major antimicrobial compounds of indigo naturalis. Isatin and tryptanthrin had no effect on tested fungi; however, indigo naturalis EA-extract had a mild effect. This anti-fungal effect may be due to other active substances in the indigo naturalis EA-extract or due to a synergism between isatin and tryptanthrin.

**Table 3:** The antimicrobial effect of isatin (Sigma) on tested microorganisms. Only the strains sensitive to the isatin are shown.

Test Strains	Quantity of isatin per disc (µg)				
	0 µg	12.5 µg	25 µg	50 µg	100 µg
*Gram-positive bacteria*					
*S. aureus*	0 ± 0 *	0 ± 0	0 ± 0	0 ± 0	34 ± 7
MRSA	0 ± 0	0 ± 0	0 ± 0	0 ± 0	38 ± 6
*S. epidermis*	0 ± 0	0 ± 0	0 ± 0	13 ± 4	57 ± 11
*S. pneumoniae*	0 ± 0	0 ± 0	0 ± 0	0 ± 0	24 ± 5
*Gram-negative bacteria*					
*E. coli*	0 ± 0	0 ± 0	0 ± 0	0 ± 0	31 ± 5
*K. pneumoniae*	0 ± 0	0 ± 0	0 ± 0	0 ± 0	28 ± 7

* The diameters of the inhibition zones (diameter of inhibition zone minus diameter of disc) were measured in mm after incubation for 24 h at the optimal temperature for the individual strains tested (see Table 1). Data are the means of triplicates ± standard deviation of a representative experiment.

**Table 4:** The antimicrobial effect of tryptanthrin (Sigma) on tested microorganisms. Only the strains sensitive to the tryptanthrin are shown.

Test Strains	Quantity of tryptanthrin per disc (µg)				
	0 µg	12.5 µg	25 µg	50 µg	100 µg
**Gram-positive bacteria**					
*S. aureus*	0 ± 0 *	53 ± 10	79 ± 11	95 ± 8	108 ± 17
MRSA	0 ± 0	0 ± 0	0 ± 0	23 ± 6	41 ± 8
*S. epidermis*	0 ± 0	124 ± 18	138 ± 28	161 ± 14	177 ± 11

* The diameters of the inhibition zones (diameter of inhibition zone minus diameter of disc) were measured in mm after incubation for 24 h at the optimal temperature for the individual strains tested (see Table 1). Data are the means of triplicates ± standard deviation of a representative experiment.

Isatin at the highest tested concentration (100 µg per disc) showed weak inhibitory effect on the growth of MRSA, *S. aureus*, *S. epidermis*, *S. pneumonia*, *E. coli*, and *K. pneumonia* (Table 3). On the other hand, tryptanthrin significantly (≥12.5 μg/disc) inhibited the growth of *S. epidermis* and *S. aureus*, but only weakly suppressed MRSA even at 100 µg per disc (Table 4). Furthermore, the inhibitory effect of tryptanthrin against the two *Staphylococcus* strains increased as the dosage increased (observed using agar diffusion and microtiter plate assays). The IC_50_ values of tryptanthrin (determined using microtiter plate assay) against the *S. aureus* and *S. epidermis* were 31 and 17 µg mL^−1^, respectively. Although indigo naturalis consists mainly of indigo blue and indirubin, both of these ingredients showed no inhibitory effect on *S. aureus* and *S. epidermis* in this study (data not shown).

We observed that the growth-inhibitory effects on *Staphylococcus* species were mainly due to trythathrin (Table 3 and Table 4). There were differences among strains: tryptathrin growth inhibition was weak against MRSA ATCC 43,000 but strong against *S. aureus* ATCC 6,538. These results are comparable to the effects of β-lactam antibiotics, however, the mechanisms by which tryptanthrin inhibits S. aureus are still unclear and need further investigation.

In previous studies, IC_50_ values of tryptanthrin in the low µg mL^−1^ range are effective against some pathogenic organisms such as *B. subtilis* [12] and *Mycobacterium tuberculosis* [13]. In this study, IC_50_ values of tryptanthrin in the low µg mL^−1^ range are effective against *S. aureus*, and *S. epidermis*. Recently, the mechanism of action of tryptanthrin has been reported to inhibit the growth of pathogens by intercalation into bacterial DNA [14]. The significant and highly selective activity of tryptanthrin against *Staphylococcus* members may be advantageous to the development of tryptanthrin-derived compounds. These compounds could be designed to specifically inhibit the growth of *Staphylococcus* species in human hosts without affecting beneficial gastrointestinal bacteria and reducing the occurence of antibiotic-associated diarrhea, which is a frequent adverse effect of antibiotic therapy [15].

Isatin derivatives are known to be associated with a broad spectrum of antibacterial and anti-inflammatory effects [16,17,18]. A series of isatin-derived metal complexes were synthesized and screened for antibacterial activity against a panel of microorganisms [19]. All the synthesized compounds showed good microbial activities with increased potency in complexes with metal ions [19]. The anti-bacterial activity of isatin was inferior to tryptanthrin in our data, although isatin alone (as high as 100 µg per disc) was able to inhibit the growth of a variety of bacteria (Table 3 and Table 4). The mechanisms of the antibacterial activity of isatin are still unclear and need further investigation.

### 2.4. The Origin of the Bioactive Compounds from Indigo Naturalis

**Figure 2:**
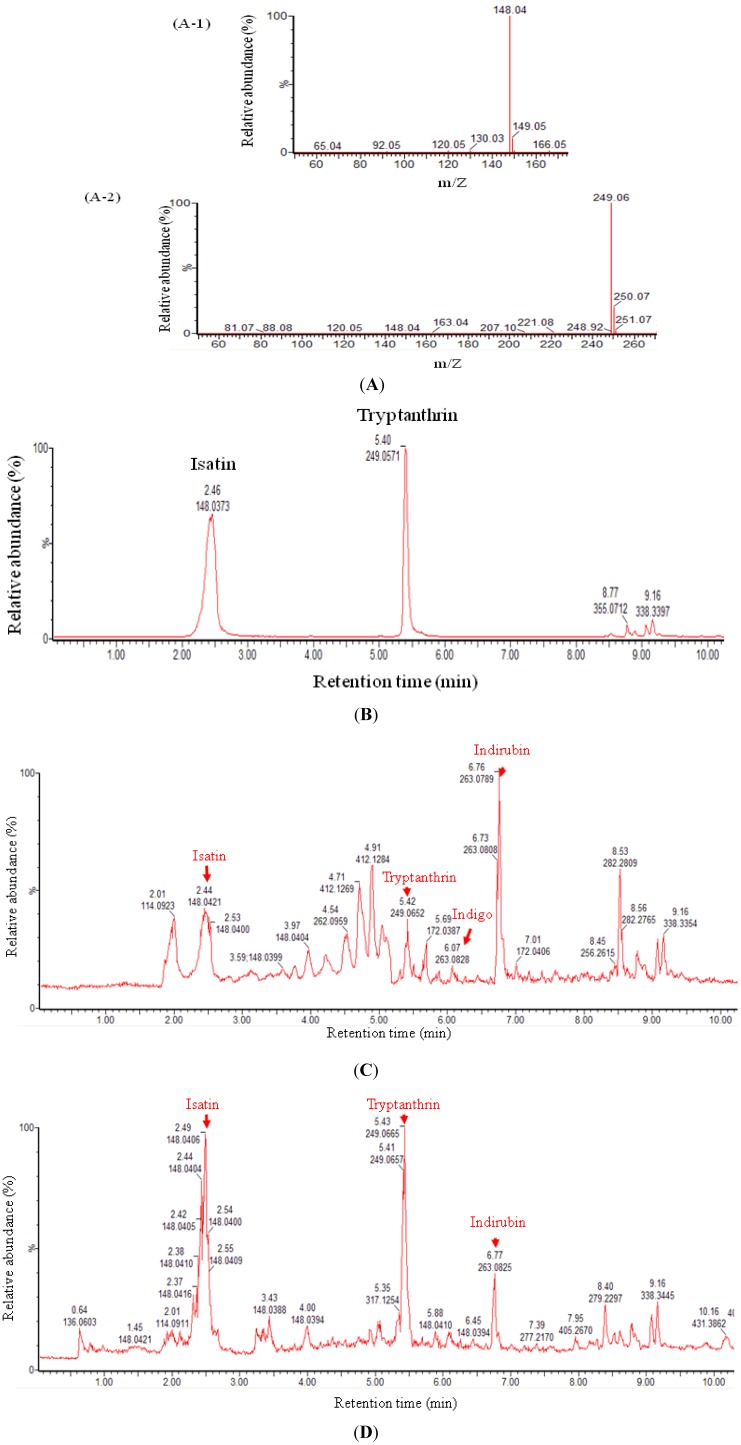
UPLC-APCI-MS analysis of the standards (isatin and tryptanthrin) and EA-extracts of indigo naturalis and *Strobilanthes formosanus* Moore. (**A**) APCI-mass spectra of isatin (A-1) and tryptanthrin (A-2) purchased from Sigma-Aldrich Chemicals; (**B**) UPLC chromatogram of the mixture (molar ratio = 1:1) of isatin and tryptanthrin purchased from Sigma-Aldrich Chemical; (**C**) UPLC total ion chromatogram of the EA-extract of indigo naturalis powder (50 µg); (**D**) UPLC total ion chromatogram of the EA-extract of the dry leaves from *S. formosanus* (50 µg).

In this study, ethyl acetate was used to extract indigo naturalis powder and the dry leaves from *Strobilanthes formosanus* Moore. UPLC-APCI-MS was applied to analyze the EA-extracts (Figure 2C,D).

Traditionally, indigo naturalis is a powder extracted from the branches and leaves of indigo-producing plants using a fermentation method and each plant can constitute itself as a drug (*Strobilanthes formosanus* Moore was used in this study). In brief, the freshly cut plants are soaked in water for several days until they decompose. During the fermentation process, microbial activities are required to decompose the plants; therefore, the possibility that the bioactive compounds might originate from microbial activities cannot be excluded.

In this study, we compared the non-polar profiles of the fresh plant leaves (Figure 2D) and indigo naturalis (Figure 2C) using UPLC-MS. The data showed that both samples contained isatin and tryptanthrin. Thus, in our case, the antimicrobial compounds (isatin and tryptanthrin) are produced directly by the plants. Note that many compounds are observed only in the indigo naturalis, but not in the fresh plant sample. These compounds are produced during the microbial fermentation process.

**Figure 3:**
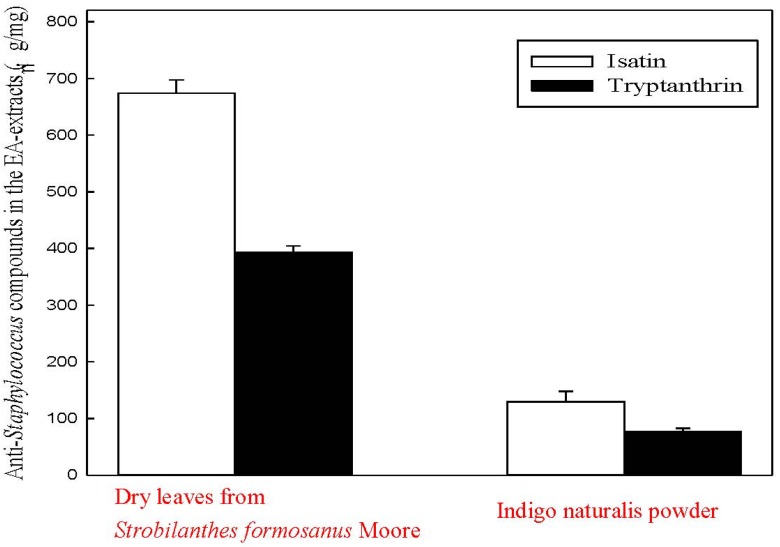
HPLC quantification of the anti-*Staphylococcus* compounds (isatin and tryptanthrin) in the EA-extracts of the indigo naturalis powder and the dry leaves from * Strobilanthes formosanus* Moore.

A metabolomics approach was applied to investigate the profiles of EA-extractable metabolites derived from indigo naturalis and its original plant, *Strobilanthes formosanus* Moore. Isatin and tryptanthrin were both present in all extracts, which suggested that both bioactive compounds originate from *Strobilanthes formosanus* Moore (Figure 2C,D). We used HPLC to quantify the two antimicrobial compounds in the EA-extracts of the dry leaves from *Strobilanthes formosanus* Moore and from the indigo naturalis powder (Figure 3). It is notable that the proportions of isatin and tryptanthrin decreased in the indigo naturalis extract (Figure 2C and Figure 3) when compared to those of the *Strobilanthes formosanus* Moore extract (Figure 2D and Figure 3).

Indirubin and indigo were also identified in the EA-extracts of the indigo naturalis powder (Figure 2C). It is worth mentioning that indirubin is one of the major compounds in the EA-extract of the dry leaves from *Strobilanthes formosanus* Moore (Figure 2D). The UPLC retention time and the APCI-spectra of indirubin and indigo are identical to those of the authentic compounds purchased from Sigma. In addition, some unidentified metabolites were present only in the EA-extract of indigo naturalis (Figure 2C). The high proportion of unidentified metabolites in indigo naturalis may be products resulting from the fermentation process of *Strobilanthes formosanus* Moore*.*

### 2.5. Discussion

This is the first report, to our knowledge, to investigate the antimicrobial activity of *Strobilanthes formosanus* Moore, one of indigo naturalis’ source plants. These *in vitro* antimicrobial tests suggest that indigo naturalis has antimicrobial effects and the results provide evidence that indigo naturalis can ameliorate psoriatic nails coexisting with onychomycosis and paronychia. However, it cannot be determined whether it: (1) directly inhibits the growth of pathogens on the nails; (2) prevents the invasion of pathogens by restoring the damaged nail structure.

The barrier function of a normal nail or the skin is that it prevents the invasion of pathogens. The main effect of indigo naturalis is that it restores the damaged nail and skin structure which, consequently, protects nails and skin from bacterial and fungal colonization. In one of our previous studies, we demonstrated that indigo naturalis can inhibit proliferation and promote differentiation in epidermal keratinocytes [20]. In addition, we also found that indigo naturalis upregulates claudin-1 expression and restores tight junction proteins in keratinocytes that help restore the impaired epidermal barrier [21].

In this study, we found MRSA was the most sensitive bacterium to indigo naturalis EA-extract among tested *Staphylococcus* strains. Neither of the bioactive ingredients from indigo naturalis, (<100 μg/disc) and tryptanthrin (<50 μg/disc), was capable of inhibiting the growth of MRSA. Therefore, the inhibitory activity of indigo naturalis EA-extract against MRSA may come from other unidentified ingredients. Currently, our lab is in the process of purifying and identifying the MRSA-inhibiting ingredients from indigo naturalis EA-extract. Our results shown in Table 2 also demonstrated that indigo naturalis EA-extract had inhibitory effects on non-dermatophytic pathogens (*A. fumigates* and *C. albicans*). However, isatin and tryptanthrin showed no effect on tested fungi. Again, some unidentified ingredients may play a role in the observed anti-fungal activity. We plan to investigate bioactive ingredients from indigo naturalis that inhibit non-dermatophytic pathogens. We expect that these results can be applicable to the treatment of non-dermatophytic onychomycosis in the future.

## 3. Experimental

### 3.1. Chemicals and Microbial Strains

The isatin and tryptanthrin used in the antimicrobial assays were obtained from Sigma (St. Louis, MO, USA). The other chemicals used were of analytical grade and were purchased from BD Diagnostics (Sparks, MD, USA), Sigma-Aldrich (St. Louis, MO, USA), Merck (Darmstadt, Germany), and Roth (Karlsruhe, Germany). The antibacterial and antifungal activities were tested against seven fungal strains including three dermatophytic pathogens (*E. floccosum* ATCC 18,397, *M. gypseum* ATCC 14,683, and *T. rubrum* ATCC 10,218) and a wide range of bacteria including human pathogens such as *K. pneumoniae* ATCC 13,883, *P. aeruginosa* ATCC 27,853, *S. aureus* ATCC 6538, MRSA ATCC 43,300, and *S. pneumoniae* ATCC 33,400 (Table 1). Tested bacterial and fungal strains were purchased from the American Type Culture Collection (ATCC; Manassas, VA, USA). The media used to cultivate these strains were prepared according to the recommended media list on the ATCC website [22].

### 3.2. The Preparation of Indigo Naturalis

The species for indigo naturalis varies in different parts of the world. *Strobilanthes formosanus* Moore, which is the species available in Taiwan, was harvested in Sansia, New Taipei City, Taiwan. The species was identified by Dr. Rong-Chi Yang, the chief of the Chinese Herbal Pharmacy at Chang Gung Memorial Hospital. The harvested leaves of *Strobilanthes formosanus* Moore were immersed in water for several days until the leaves were decomposed by microbial activities. After that, lime was the only substance added to the suspension to precipitate indigo naturalis. The chemicals included in indigo naturalis are different from *Strobilanthes formosanus* Moore due to the fermentation process.

Indigo naturalis powder prepared from *Strobilanthes formosanus* Moore was purchased from Guang Sheng Trading (Taipei, Taiwan). The purity was ascertained by Dr. Yann-Lii Leu. A voucher specimen (SF-1) was deposited in the herbarium of Chang Gung University, Taoyuan, Taiwan.

### 3.3. Preparation of Ethyl Acetate (EA) Extracts of *Strobilanthes formosanus* Moore and Indigo Naturalis

The leaves of *Strobilanthes formosanus* Moore were dried in an oven (40 °C) for three days. Twenty-five g of the dried *Strobilanthes formosanus* Moore leaves were extracted with ethyl acetate (EA, 300 mL) at 40 °C for one hour. Indigo naturalis (25 g) was also extracted by the same procedure. The EA-extractable compounds of the two samples were then separated from the residue by centrifugation (12,000 *×g*, 20 min) at 15 °C. The supernatant was recovered and evaporated to dryness under reduced pressure and stored at −20 °C for bioassays.

### 3.4. Agar Diffusion Assay

To obtain fresh pre-cultures, the tested strains were first cultivated in a 60 mL medium in 250 mL Erlenmeyer flasks. The media and incubation temperature for the different bacterial and fungal strains are summarized in Table 1. Incubation was performed under aerobic conditions with shaking (180 rpm) and the growth of tested strains was determined by measuring the optical density (OD) at 600 nm. Cultures in the exponential growth phase (OD_600nm_ reached 0.5~0.8; with an optical path of 1 cm) were diluted to OD_600nm_ = 0.1, and immediately used for the following antimicrobial assays. The antimicrobial activities of EA extracts were tested by an agar diffusion assay described by Finn [23] with minor modifications because many fungal strains, especially dermatophytes, cannot grow evenly in liquid media. The discs (6 mm diameter) containing bioactive compounds (10, 25, 50, or 100 µg per disc) were placed onto agar plates seeded with tested strains. Antimicrobial activities of bioactive compounds were tested in triplicate. The diameters of the inhibition zones (diameter of inhibition zone minus diameter of disc) were measured in mm after incubation at optimal temperature for 24 h (see Table 1). For internal controls, authentic antibiotics (kanamycin, penicillin, and tetracycline for the test bacterial strains, and cyclohexamide for the test fungal strains) were used.

### 3.5. Microtiter Plate Assay

The median inhibitory concentration (IC_50_) values of bioactive compounds (isatin and tryptanthrin) of indigo naturalis against *Staphylococcus* strains were determined using a 96-well microtiter plate assay as described by Skyttä and Mattila-Sandholm [24] with minor modifications. To determine IC_50_ values of the active compounds, serial two-fold dilutions were prepared in the range of 1.6~100 µg mL^−1^ (final concentration). IC_50_ values were expressed as the dry mass (µg) of bioactive compounds required to inhibit 50% of cell growth (OD_600nm_) of tested organisms grown in 100 µL of inoculated medium. For determination of IC_50_ values, at least three independent experiments, each with three replicates, were conducted. For internal controls, authentic antibiotics (kanamycin, penicillin, and tetracycline) for the test bacterial strains were used [24].

### 3.6. Gel Permeation Chromatography (GPC)

The bioactive compounds of indigo naturalis EA-extracts were first separated on a 385-mL Sephadex LH-20 column (55 by 3 cm; Pharmacia Biotech, Uppsala, Sweden). The resin was equilibrated with two bed volumes of methanol. The active fraction was loaded to the column, and was eluted with methanol at a flow rate of 1 mL min^−1^. Bioactive fractions were collected and evaporated to dry. One mL of ethanol was used to re-dissolve the residue.

### 3.7. Thin Layer Chromatography (TLC)

The bioactive compounds of indigo naturalis were then separated on silica gel aluminum TLC plates (Silica gel 60 F_254_, thickness, 0.2 mm, 20 by 20 cm; Merck). The following developing solvent system was used: dichloromethane-ethyl acetate-ethanol (14:4:1, v/v). The bioactive compounds were visualized under UV light at 254 nm or visualized by spraying the TLC plates with 30% (v/v) H_2_SO_4_.

### 3.8. High-Performance Liquid Chromatography (HPLC)

A reverse-phase Hitachi HPLC system was used for the final separation of bioactive compounds of indigo naturalis. An analytical RP-C_18_ column (Luna C18 (2), 5 μm, 150 by 4.6 mm; Phenomenex, Torrance, CA, USA) was used with flow rate of 0.4 mL min^−1^ at room temperature. The mobile phase was 100% (v/v) methanol. Bioactive compounds were detected with a UV detector (L-2400; Hitachi, Schaumburg, IL, USA) at 240 nm. In addition, HPLC was used for the quantification of isatin and tryptanthrin present in the ethyl acetate extracts of the dry leaves from *Strobilanthes formosanus* Moore and from the indigo naturalis powder. The quantification of isatin (detected at 240 nm) and tryptanthrin (detected at 250 nm) was calculated from their respective peak areas using a standard curve of individual standards. The *R*^2^ values for the standard curves were >0.98. Data are averages of three measurements.

### 3.9. UV-VIS Spectroscopy

Standards and HPLC-purified bioactive compounds were dissolved in acetonitrile in the range of 5~10 μg mL^−1^. UV absorption spectra of these compounds were obtained using a U-1900 UV/VIS spectrometer (Hitachi, Japan).

### 3.10. Ultra-Performance Liquid Chromatography-Atmospheric Pressure Chemical Ionization-Mass Spectrometry (UPLC-APCI-MS)

The EA-extractable samples (50 µg for each), standards (isatin and tryptanthrin), and HPLC-purified bioactive compounds were analyzed by UPLC-MS with UPLC coupled to an APCI-mass spectrometer. Mass spectral data were obtained using a Micromass ZQ quadruple mass spectrometer (Waters, Milford, MA, USA) equipped with a standard APCI source operating in the positive ion mode. Separation was achieved on a reversed-phase C_18_ column (Acquity UPLC^TM^ BEH C18, 1.7 μm, 100 × 1.0 mm; Waters) with a flow rate of 0.1 mL min^−1^ at 35 °C (column oven temperature). The mobile phase consisted of a mixture of two solvents: solvent A (1% (v/v) acetonitrile containing 0.1% formic acid to enable good ionization in the APCI) and solvent B (methanol containing 0.1% formic acid). Separation was achieved with a linear gradient of solvent B from 0% to 100% in 10 min. In the APCI-MS analysis, the temperature of the ion source was maintained at 100 °C. Nitrogen desolvation gas was set at a flow rate of 500 L h^–1^ and the probe was heated to 400 °C. Nitrogen was used as the APCI carrier gas. The corona current was maintained at 20 μA, and the electron multiplier voltage was set to 650 eV. The parent scan was in the range of 50~500 (*m/z*).

### 3.11. NMR Spectroscopy

The ^1^H- and ^13^C-NMR spectra were recorded at 27 °C with a Bruker Avance-400 FT-NMR spectrometer. Chemical shifts (δ) were recorded and are shown as ppm values with deuterated acetone (99.5%, ^1^H: δ = 7.26 ppm; ^13^C: δ = 77.0 ppm) as the solvent and internal reference.

## 4. Conclusions

This study provides the first evidence of antimicrobial activities of indigo naturalis, originating from *Strobilanthes formosanus* Moore, on *Staphylococcus* spp. and non-dermatophytic onychomycosis pathogens. Using *S. aureus* and *S. Epidermis* as the model organisms for bioassay, we identified tryptanthrin as the major bioactive component with inhibitory effect on *S. aureus*. The data of this study suggest that in psoriatic nail coexisting with onychomycosis and paronychia ameliorated by indigo naturalis, antimicrobial activity and repairment of the damaged nail structure may be a simultaneous and synergistic process. These results should improve understanding of the pharmacologic mechansims of indigo naturalis in treating psoriatic nails coexisting with onychomycosis and paronychia.

## References

[1] Mak N.K., Leung C.Y., Wei X.Y., Shen X.L., Wong N.S., Leung K.N., Fung M.C. (2003). Inhibition of RANTES expression by indirubin in influenza virus-infected human bronchial epithelial cells. Biochem. Pharmacol..

[2] Fatima I., Ahmad I., Anis I., Malik A., Afza N. (2007). Isatinones A and B, new antifungal oxindole alkaloids from *Isatis costata*. Molecules.

[3] Lin Y.K., Wong W.R., Chang Y.C., Chang C.J., Tsay P.K., Chang S.C., Pang J.H.S. (2007). The efficacy and safety of topically applied indigo naturalis ointment in patients with plaque-type psoriasis. Dermatology.

[4] Lin Y.K., Chang C.J., Chang Y.C., Wong W.R., Chang S.C., Pang J.H.S. (2008). Clinical assessment of patients with recalcitrant psoriasis in a randomized, observer-blind, vehicle-controlled trial using indigo naturalis. Arch. Dermatol..

[5] Lin Y.K. (2011). Indigo naturalis oil extract drops in the treatment of moderate to severe nail psoriasis: A small case series. Arch. Dermatol..

[6] Lin Y.K., See L.C., Chen J.L., Leu Y.L., Tsou T.C., Shen Y.M. (2011). Treatment of psoriatic nails with indigo naturalis oil extract: A non-controlled pilot study. Dermatology.

[7] Kaçar N., Ergin S., Ergin C., Erdogan B.S., Kaleli I. (2007). The prevalence, aetiological agents and therapy of onychomycosis in patients with psoriasis: A prospective controlled trial. Clin. Exp. Dermatol..

[8] Zisova L., Valtchev V., Sotiriou E., Gospodinov D., Mateev G. (2012). Onychomycosis in patients with psoriasis - a multicentre study. Mycoses.

[9] Chi C.C., Wang S.H., Chou M.C. (2005). The causative pathogens of onychomycosis in southern Taiwan. Mycoses.

[10] Brook I. (2007). The role of anaerobic bacteria in cutaneous and soft tissue abscesses and infected cysts. Anaerobe.

[11] Fitzpatrick T.B., Johnson R.A., Wolff K., Suurmond D. (2001). Color Atlas and Synopsis of Clinical Dermatology: Common and Serious Diseases.

[12] Schindler F., Zahner H. (1971). Metabolic products of microorganisms. 91. Tryptanthrin, a tryptophan derived antibiotic from *Candida* lipolytica. Arch. Microbiol..

[13] Mitscher L.A., Baker W. (1998). Tuberculosis: A search for novel therapy starting with natural products. Med. Res. Rev..

[14] Bandekar P.P., Roopnarine K.A., Parekh V.J., Mitchell T.R., Novak M.J., Sinden R. (2010). Antimicrobial activity of tryptanthrins in *Escherichia coli*. J. Med. Chem..

[15] Ayyagari A., Garg J.A.A. (2003). Antibiotic associated diarrhea: Infectious causes. IndianJ. Med. Microbiol..

[16] Verma R.S., Nobles W.L. (1975). Antiviral, antibacterial and antifungal activities of isatin-N-Mannich bases. J. Pharm. Sci..

[17] Kupinić M., Medić-Sarić M., Movrin M., Maysinger D. (1979). Antibacterial and antifungal activities of isatin N-Mannich bases. J. Pharm. Sci..

[18] Alagarsamy V., Meena S., Revathi R. (2004). Anti-HIV, antibacterial and antifungal Activities of some 2,3-disubstituted quinazolin-4(3H)-ones. Indian J. Pharm. Sci..

[19] Chohan Z.H., Pervez H., Rauf A., Khan K.M., Supuran C.T. (2004). Isatin-derived antibacterial and antifungal compounds and their transition metal complexes. J. Enzyme Inhib. Med. Chem..

[20] Lin Y.K., Leu Y.L., Yang S.H., Chen H.W., Wang C.T., Pang J.H.S. (2009). Anti-psoriatic effects of indigo naturalis on the proliferation and differentiation of keratinocytes with indirubin as the active component. J. Dermatol. Sci..

[21] Lin Y.K., Chen H.W., Leu Y.L., Yang Y.L., Fang Y., Hwang T.L. (2013). Indigo naturalis upregulates claudin-1 expression in human keratinocytes and psoriatic lesions. J. Ethnopharmacol..

[22] American Type Culture Collection (ATCC). http://www.atcc.org/.

[23] Finn R.K. (1959). Theory of agar diffusion methods for bioassay. Anal. Chem..

[24] Skyttä E., Mattila-Sandholm T. (1991). A quantitative method for assessing bacteriocins and other food antimicrobials by automated turbidometry. J. Microbiol. Methods.

